# Intravascular papillary endothelial hyperplasia within the uterine myometrium: a case report and literature review

**DOI:** 10.3389/fonc.2026.1808753

**Published:** 2026-05-20

**Authors:** Li-li Wei, Li-xing Zhang, Xiang-lan Mo

**Affiliations:** Department of Pathology, People’s Hospital of Guangxi Zhuang Autonomous Region, Nanning, China

**Keywords:** clinicopathological characteristics, histopathology, intravascular papillary endothelial proliferation, uterine myometrium mass, vascular lesion

## Abstract

Intravascular papillary endothelial hyperplasia (IPEH) is a rare vascular lesion that can histologically mimic malignant vascular tumors, particularly angiosarcoma, often leading to misdiagnosis and overtreatment. IPEH involving the uterine myometrium is a novel finding. We describe an 83-year-old postmenopausal woman presenting with a 6-day history of spontaneous vaginal bleeding. Pelvic examination revealed a patent vagina with minimal dark red blood and an enlarged, anteverted uterus. Pelvic magnetic resonance imaging showed a well-demarcated, heterogeneous intramural lesion (6.0 × 5.5 × 5.0 cm) in the right anterior uterine wall. Based on comprehensive imaging findings and clinical presentation, the preoperative differential diagnosis included possible sarcomatous transformation of a uterine leiomyoma versus endometrial carcinoma. The patient underwent total laparoscopic hysterectomy, bilateral salpingo-oophorectomy, and pelvic adhesiolysis. Gross examination revealed a well-circumscribed intramural cystic lesion. Histopathology demonstrated a collagenous cyst wall with residual smooth muscle, luminal organizing thrombus, and papillary structures lined by plump endothelial cells. Immunohistochemistry showed strong positivity for cluster of differentiation (CD)31 and ETS-related gene, focal positivity for CD34 and D2-40, negative hormone receptors (estrogen receptor/progesterone receptor), and a Ki-67 index of 20%. Histopathological examination confirmed a diagnosis of IPEH, a rare benign entity characterized by reactive endothelial proliferation. Complete surgical excision resulted in resolution of vaginal bleeding. At an 18-month telephone follow-up the patient was asymptomatic, and had received no adjuvant therapy, and pelvic ultrasonography demonstrated no evidence of recurrent disease. In conclusion, IPEH cannot be reliably distinguished from malignant vascular or uterine neoplasms based on preoperative clinical, imaging, or gross findings. Accurate diagnosis of this rare entity is contingent upon histopathological examination and immunohistochemical analysis. Awareness that IPEH can occur within the uterine corpus may facilitate its inclusion in preoperative differential diagnosis, thereby potentially improving diagnostic precision and alleviating patient anxiety associated with the suspicion of malignancy.

## 1 Introduction

Intravascular papillary endothelial hyperplasia (IPEH) is a rare vascular lesion that predominantly arises in the head, trunk, and extremities Histopathologically, IPEH can resemble other benign or malignant vascular tumors, particularly when lesions occur in atypical sites or reach a significant size. This overlap often leads to misdiagnosis as angiosarcoma and may result in overtreatment. A systematic literature search was conducted to identify previously reported cases of uterine IPEH. PubMed/MEDLINE, Embase, Web of Science, China National Knowledge Infrastructure (CNKI), and the Chinese Medical Journals Full-Text Database were searched from database inception to January 1, 2026, with no date restrictions applied. The following search terms were used: (‘intravascular papillary endothelial hyperplasia’ OR ‘Masson’s tumor’ OR ‘Masson tumor’ OR ‘IPEH’) AND (‘uterine’ OR ‘uterus’ OR ‘myometrium’ OR ‘myometrial’). The reference lists of retrieved articles were manually screened for additional relevant cases. Only case reports or case series describing IPEH involving the uterine corpus or myometrium were included; cases limited to extrauterine sites were excluded. To the best of our knowledge, this search confirmed that the present case is the first reported instance of IPEH confined to the uterine myometrium. Reporting such a case can provide insights into its clinical and pathological features and may help clinicians minimize misdiagnosis. Here we present a case of primary IPEH arising in an 83-year-old woman who presented with irregular vaginal bleeding. The diagnosis was confirmed via histopathological examination, and immunohistochemical analysis. Through this report and a review of the relevant literature, we aim to enhance understanding of this rare lesion and highlight considerations for accurate diagnosis and management.

## 2 Case presentation

An 83-year-old woman, postmenopausal for 33 years, presented with a 6-day history of spontaneous vaginal bleeding. Pelvic examination revealed a patent vagina with minimal dark red blood and an atrophic cervix. The uterus was anteverted and enlarged, with no palpable adnexal masses and was non-tender. Preoperatively the premenopausal Risk of Ovarian Malignancy Algorithm (ROMA) score was mildly elevated, but serum tumor markers including carbohydrate antigen 12-5 (CA12-5), human epididymis protein 4 (HE4), carbohydrate antigen 19-9 (CA19-9), and squamous cell carcinoma antigen (SCCA) were within their respective reference ranges. Preoperative serum tumor marker levels and their corresponding clinical interpretations are summarized in **Table 1**. Collectively, these preoperative findings did not provide serological evidence suggestive of malignancy.

**Table 1:** Preoperative serum tumor marker profile.

Biomarker (abbreviation)	Measured value	Institutional reference range	Clinical interpretation
CA12-5	14.10 U/mL	0−35 U/mL	Normal. Although sensitivity for early-stage ovarian malignancy is limited, the result does not support the presence of significant peritoneal dissemination or advanced epithelial disease.
HE4	88.5 pmol/L	0−132 pmol/L	Within normal limits. HE4 levels are typically unaffected by endometriosis or menstrual status; therefore, a normal value reinforces the low likelihood of epithelial ovarian malignancy in conjunction with the normal CA125 and low ROMA score.
CA19-9	9.25 U/mL	0.0−34.0 U/mL	Unremarkable. Provides no serological evidence of pancreaticobiliary pathology.
SCCA	0.59 ng/mL	0.0−2.7 ng/mL	Normal. Suggests a lack of clinically significant squamous cell carcinoma of cervical, pulmonary, esophageal, or head and neck origin.
ROMA score^†^	Pre: 23.78% Post: 18.38%	Pre: < 11.4% Post: < 29.9%^‡^	Premenopausal elevation reflects benign CA125 interference; postmenopausal normalization suggests a lack of occult malignancy.

^†^ ROMA score calculated based on CA125, HE4, and menopausal status in accordance with the manufacturer’s algorithm.

^‡^ Reference cut-off varies by assay platform; values shown are illustrative for a specific commercial assay.

**Figure 1:**
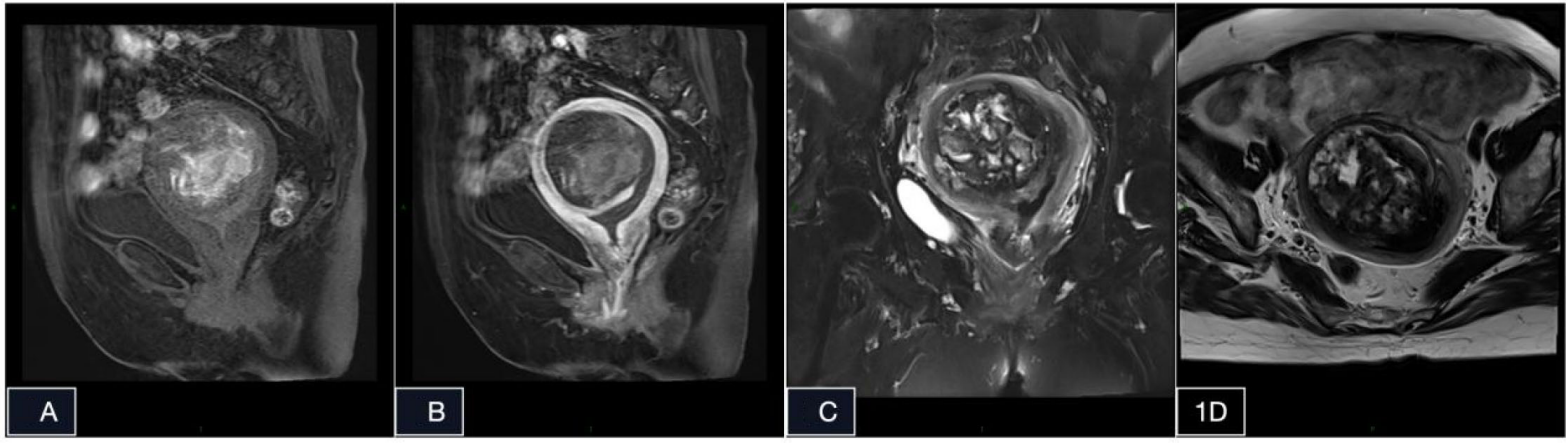
Precontrast T1-weighted **(A)**,Postcontrast T1-weighted **(B)**, Precontrast T1-weighted **(C, D)**. Preoperative MRI showing a 6 cm × 5.5 cm × 5 cm tissue mass with heterogeneous signal which was identified in the anterior right wall of the uterus, predominantly exhibiting high signals on both T1 and T2-weighted imaging. Additionally, a few patchy delayed enhancements were observed during enhanced scanning.

Pelvic magnetic resonance imaging (MRI) revealed a well-demarcated, heterogeneous intramural lesion in the right anterior uterine wall measuring 6.0 × 5.5 × 5.0 cm. The lesion showed T1/T2 hyperintensity with peripheral nodular enhancement and abutment of the endometrium, whereas the junctional zone remained intact (**Figure 1**). Imaging findings suggested possible malignant transformation of a leiomyoma.

The patient had a significant past medical history notable for uterine adenomyosis, sequelae of pelvic inflammatory disease, hypertension, and diabetes mellitus. Blood pressure was maintained within normal limits with regular oral administration of extended-release nifedipine. The patient had a history of diabetes mellitus for over 10 years, managed with subcutaneous insulin injections; however, glycemic control remained suboptimal. She reported no history of uterine malignancy, prior surgery, or trauma.

Preoperative imaging raised a concern of possible malignant transformation of a leiomyoma, but endometrial carcinoma could not be excluded by the gynecologist. Given the high clinical suspicion of malignancy, a multidisciplinary discussion was held. Considering the patient’s age (>80 years) and adherence to current guidelines for uterine malignancies, after preoperative multidisciplinary discussion the planned surgical approach was total laparoscopic hysterectomy with bilateral salpingo-oophorectomy. Under general anesthesia, pneumoperitoneum was established and a laparoscopic exploration was performed. Intraoperative findings included an enlarged uterus compatible with a gestational age of approximately 8 weeks, measuring approximately 9 × 9 × 8 cm, with a smooth serosal surface and slight bulging of the right anterior wall. Filmy adhesions were noted between the posterior uterine wall, the posterior abdominal wall, and the mesentery. Both ovaries and fallopian tubes appeared grossly unremarkable. Following thorough assessment of the pelvic anatomy, and considering the patient’s advanced age, anticipated tolerance of prolonged anesthesia, and potential postoperative morbidity, a decision was made intraoperatively to proceed with total laparoscopic hysterectomy, bilateral salpingo-oophorectomy, and pelvic adhesiolysis, and all excised tissues were submitted for histopathological analysis. Notably, the surgical complexity was comparatively less than that typically encountered in radical procedures for confirmed malignancies such as endometrial carcinoma. The total operative time was 82 minutes, and the procedure was completed uneventfully. The patient remained hemodynamically stable throughout, with an estimated intraoperative blood loss of 50 mL and a urine output of 100 mL of clear urine. She was returned to the ward in a stable condition, and no immediate postoperative complications were observed.

Gross examination showed a well-circumscribed, intramural cystic lesion measuring 5.5 × 4.5 × 4 cm with a shaggy, blood-adherent inner surface.

**Figure 2:**
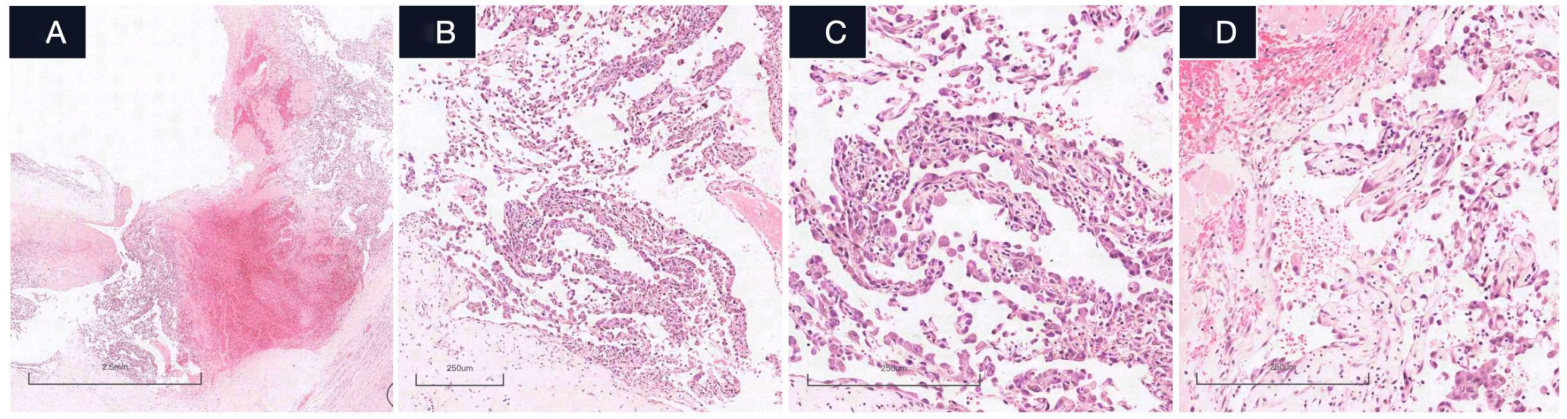
Histological microphotographs of IPEH(hematoxylin-eosin staining). **(A)** Low-power view demonstrates a cystically dilated vessel filled with extensive thrombus formation. Multiple irregular papillary structures are observed arising from the vessel wall and lining the periphery of the thrombus. (original magnification ×20). Scale bar = 2.5 mm. **(B)** showing that the dilated vascular lesion is lined with a single flat vascular endothelial cell. (original magnification ×100). Scale bar = 250 μm. **(C, D)** High-power view showing that intravascular papillary proliferation of plump endothelial cells with hyalin cores or fibrous tissue core, the chromatin in the nucleus is deeply stained, and no clear mitotic figures are observed. Note the presence of increased inflammatory cell infiltration within some of the papillary cores. (original magnification ×200). Scale bar = 250 μm.

**Figure 3:**
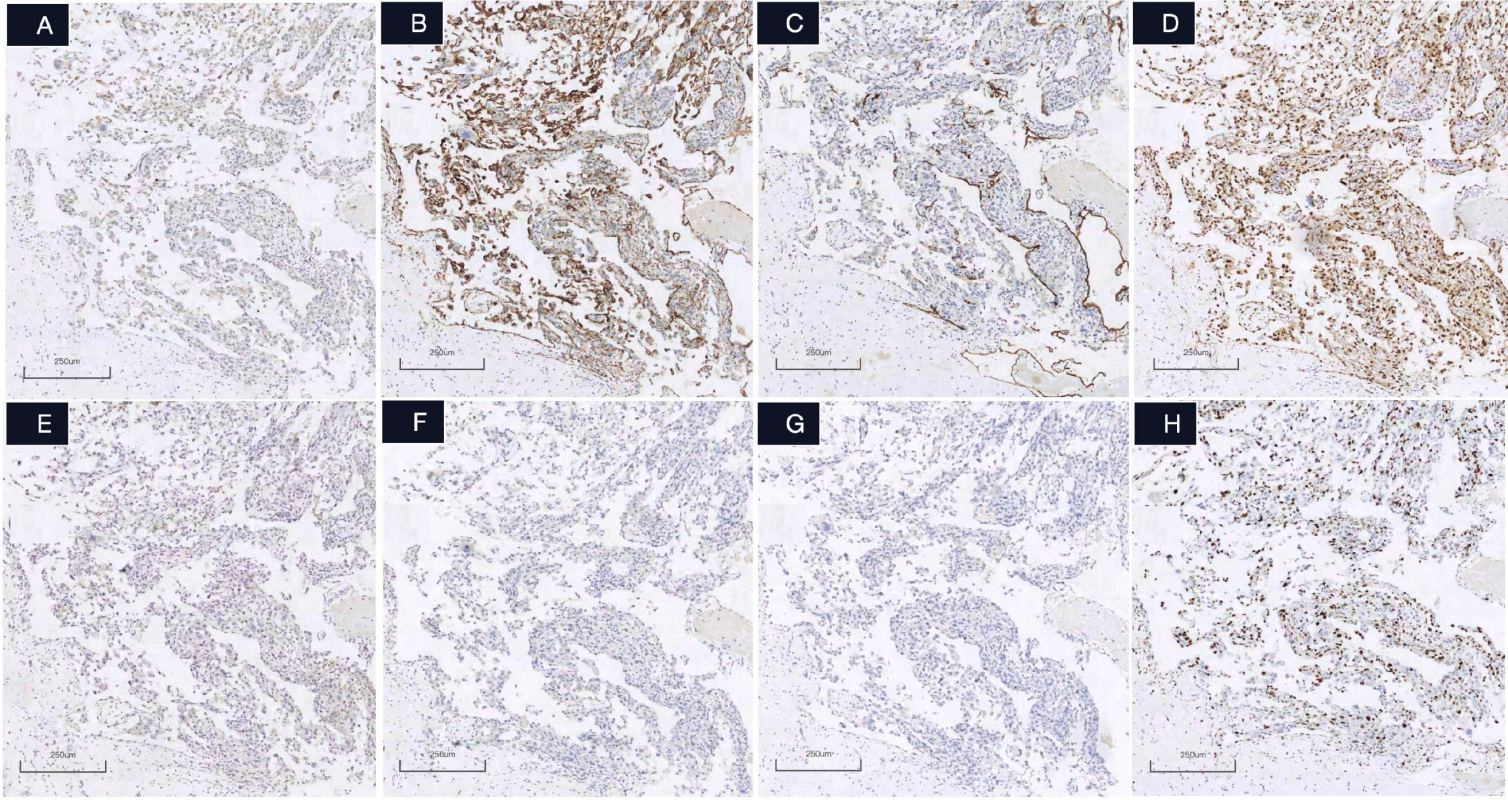
**(A–H)** (IHC). Immunostaining microphotographs of IPEH. Immunohistochemical reaction with desmin(4A) show the residual smooth muscle tissue of the vascular wall,CD31(4B) and Erg (4D) Diffuse strong membranous staining in both vascular walls and papillary lining endothelial cells (confirming endothelial differentiation),CD34(4C) Partial endothelial immunoreactivity and D4-20(4E) Focal weak positivity in endothelial cells, while ER(4F) and PR(4G) were Completely negative in endothelial cells, ki67(4H) Approximately 20% proliferation index in endothelial cells (note: some positive cells represent infiltrating inflammatory cells). (original magnification ×100). Scale bar = 250 μm.

Microscopic examination revealed three diagnostically significant components: (1) a collagenous cyst wall harboring residual smooth muscle fascicles, (2)luminal organizing thrombus with hyalinization and fibroblastic proliferation, and (3) numerous papillary structures lined by a single layer of plump endothelial cells (**Figures 2A–D**). These papillae displayed characteristic hyalinized cores containing sparse lymphocytes, with complete absence of necrosis or significant mitotic activity (<1/50 high-power fields). Immunohistochemistry (**Figure 3**) demonstrated strong diffuse positivity for cluster of differentiation (CD)31 and ETS-related gene (ERG), focal reactivity for CD34 and D2-40, negative hormone receptors (estrogen receptor/progesterone receptor), and a Ki-67 proliferation index of 20%. The desmin+/elastic fiber+ cyst wall architecture, combined with these features, definitively confirmed the diagnosis of IPEH within a degenerated vascular malformation.

After complete surgical excision without adjuvant therapy, the patient’s symptoms resolved promptly. We conducted telephone follow-up for over 18 months postoperatively. The patient reported that she had returned home for rest and attended gynecologic outpatient visits every 6 months. Routine gynecologic examination revealed no vaginal bleeding, discharge, or palpable masses. Pelvic color Doppler ultrasound performed at each visit showed no abnormal masses. The patient reported no pain or other symptoms. Collectively, these follow-up findings demonstrated no clinical or radiographic evidence of recurrence, confirming a sustained disease-free status.

## 3 Discussion

IPEH is a benign vascular proliferation accounting for 2–4% of cutaneous/soft tissue vascular tumors (1). Clinically, it presents as slow-growing masses; its radiologic features (heterogeneous enhancement on computed tomography [CT]/MRI) (2) and histologic mimicry of angiosarcoma necessitate a definitive diagnosis through immunohistochemical confirmation (CD31+/ERG+ with variable D2-40). Epidemiologic studies demonstrate a peak incidence between the ages of 30 and 40 years, with a slight female predominance consistently observed across multiple case series. These suggest potential hormonal influences on disease pathogenesis (3, 4). The anatomic distribution typically involves the head, neck or extremities, whereas visceral cases are rare (such as stomach (5), brain (6), and mandible (7). The involvement of the female genital tract is extremely rare. For instance, only one cervical case (8) has been reported, and no prior uterine examples exist, apart from our observed lesion measuring 5.5 cm—significantly larger than the typical size of less than 2 cm (9), although outliers of up to 10 cm (10) have been documented—in this case. This lesion exhibited hemorrhage and irregular borders grossly suspicious for malignancy.

The imaging presentation of IPEH on CT and MRI frequently demonstrates enhancement patterns bearing remarkable resemblance to malignant vascular neoplasms (11), resulting in substantial diagnostic ambiguity during preoperative evaluation.

Pathogenetically, while Masson initially proposed a neoplastic origin, Clearkin and Enzinger’s reclassification as a thrombus-driven reactive process (12) aligns with existing models of trauma-induced (7) or drug-mediated (olanzapine/STAT3) (13) endothelial activation via fibroblast growth factor-β (FGF-β) autocrine loops (14). Current understanding identifies dual activation pathways in IPEH development: (i) mechanical induction through localized vascular injury or developmental anomalies initiates perivascular inflammatory cascades, where the infiltrating macrophages secrete basic FGF-β, triggering IPEH development through dual mechanisms:inducing thrombosis via fibrin deposition and stimulating endothelial proliferation. Crucially, the proliferating endothelial cells release additional FGF-β, thereby activating a positive feedback loop that perpetuates lesion growth (14) (ii) Recent studies reveal an association between prolonged use of second-generation antipsychotics (particularly olanzapine) and IPEH occurrence (13), mediated through STAT3 upregulation-induced angiogenesis.

The present patient exhibited a constellation of comorbid conditions that, while not causally linked to IPEH in the existing literature, may represent a confluence of factors that collectively favor the development of this rare lesion. Specifically, long-standing hypertension is associated with endothelial injury and altered vascular hemodynamics—a mechanism circumstantially supported by a prior report documenting IPEH hemorrhage precipitated by an acute hypertensive crisis (15). Poorly controlled diabetes mellitus is a well-established prothrombotic state that may further predispose to local thrombus formation. Uterine adenomyosis is characterized by a chronic inflammatory milieu with local elaboration of angiogenic mediators, including vascular endothelial growth factor (VEGF). Notably, tumor-like intravascular stromal proliferations have been documented in adenomyotic foci (16), suggesting that the local uterine environment in such patients may be permissive of reactive endothelial changes. It must be emphasized, however, that the interplay proposed herein—whereby systemic vascular comorbidities synergize with a localized inflammatory niche to promote IPEH development—remains entirely speculative, as no studies to date have directly investigated this hypothesis. Further investigation is warranted to elucidate whether such comorbid conditions may predispose to or modulate the clinical course of IPEH.

Microscopically, IPEH is characterized by six diagnostic features: (1) exclusive intravascular growth, (2) papillary fronds with fibrinoid cores or hyalinized collagenous cores, (3) monolayer endothelial lining composed of cytologically bland cells without significant pleomorphism or pathological mitoses, (4) absence of coagulative tumor necrosis, (5) non-infiltrative growth pattern (17), and (6) association with intralesional thrombosis. The diagnosis of IPEH principally relies on two pathognomonic features: the formation of intravascular papillary structures lined by a continuous monolayer of hypertrophic endothelial cells, and the consistent association with organizing thrombus. Our case exhibited all diagnostic hallmarks, including large vascular channels lined by hypertrophic endothelial cells, extensive mural hyalinization with residual smooth muscle, mixed thrombus formation, and stromal hyalinized collagen with patchy lymphocytic infiltration. Immunohistochemistry demonstrated diffuse CD31, ERG, and factor VIII expression, with focal CD34 and D2–40 positivity; desmin highlighted residual vascular smooth muscle. The elevated Ki-67 proliferative index (20% vs. typical <10% in extragenital IPEH) (2, 7) observed in this uterine case likely reflects tissue-specific endocrine influences, as substantiated by two key findings:the uterine corpus’s hormonally responsive microenvironment, and concordance with Susini T et al. (8) documentation of pregnancy-associated IPEH growth acceleration and hormone receptor overexpression. Although ER and PR were negative in our case, the proliferative endothelial cells exhibited reactive and reparative changes without significant atypia, mitotic activity, or necrosis. The elevated proliferative index may be associated with the heightened proliferative activity inherent to the reparative hyperplasia itself. Furthermore, microscopic examination revealed no histomorphological features consistent with endometrial carcinoma. Glandular or nested architectures were absent. Most notably, neither cytologic atypia nor architectural disorganization was identified, and there was no evidence of invasive growth. Immunohistochemical staining demonstrated positivity for vascular markers, including CD31, CD34, and ERG, a profile that effectively excludes a diagnosis of endometrial carcinoma.

Critical diagnostic distinctions exist between IPEH and other vascular lesions:(1)angiosarcoma demonstrates infiltrative growth with multilayered, markedly atypical endothelial cells unlike with IPEH’s monolayer benign endothelium; (2) Dabska tumor (18)exhibits characteristic hobnail morphology and most cases demonstrate VEGFR-3 expression, a key diagnostic marker for distinguishing Dabska tumor from other vascular lesions, absent in IPEH; (3) arteriovenous malformations display disorganized thick-walled vessels that may secondarily develop IPEH-like changes; (4)organizing hematomas lack intact vascular walls; and (5) conventional hemangiomas typically lack extensive hyalinized papillary structures. However, mixed lesions demonstrating co-existing hemangioma and IPEH components have been documented.

The present patient’s sole clinical manifestation was spontaneous postmenopausal vaginal bleeding. MRI revealed a well-circumscribed, heterogeneous lesion within the uterine myometrium, exhibiting hyperintensity on both T1-weighted and T2-weighted sequences and peripheral nodular enhancement. Taken together with the patient’s age and clinical presentation, these findings raised a strong preoperative suspicion of endometrial carcinoma. Consequently, definitive diagnosis remained contingent upon histopathological evaluation. Notably, during pathological examination of the surgical specimen, abundant intralesional thrombi were identified. This observation suggests that even if preoperative endometrial curettage or biopsy had been performed, the paucity of viable lesional tissue—owing to the extensive thrombotic component—might have precluded retrieval of adequate diagnostic material, thereby potentially yielding an inconclusive or misleading result. We therefore contend that in such cases, complete surgical excision of the lesion offers the most reliable route to an accurate histopathological diagnosis. Furthermore, the deceptive preoperative clinical and radiographic presentation in this case underscores the importance of maintaining a broad differential diagnosis. When encountering similar patients in the future, consideration should be extended beyond malignant entities to include rare benign lesions such as IPEH.

Complete surgical excision with histologically negative margins is the gold-standard treatment for IPEH. Although the prognosis is favorable, one study of 91 cases demonstrated (3) a 10% recurrence rate. The principal factors contributing to IPEH recurrence included histologically positive resection margins (19), inadequate surgical clearance (<5 mm from the lesion periphery) (20) and coexistence with other vascular neoplasms. In complex cases (21), pharmacological interventions with interferon-α and the mammalian target of rapamycin inhibitor sirolimus can achieve disease stabilization in IPEH.

## 4 Conclusions

In this case, IPEH presented as a sizable intramyometrial lesion, an exceptionally uncommon occurrence. Preoperative imaging studies did not provide a definitive characterization of the mass, and its gross morphological features contributed to diagnostic uncertainty. The diagnosis was confirmed through histopathological examination, supported by immunohistochemical profiling. Following complete surgical resection, the patient’s vaginal bleeding and discharge resolved completely, and no recurrence was observed during the 18-month clinical follow-up.

Histologically, IPEH is frequently misdiagnosed as a capillary hemangioma or even angiosarcoma, underscoring its diagnostic complexity. Thus, a multidisciplinary approach that integrates histomorphology, clinical presentation, imaging findings, and immunohistochemical markers is essential to ensure accurate diagnosis and guide optimal clinical management.

## Data Availability

The original contributions presented in the study are included in the article/supplementary material. Further inquiries can be directed to the corresponding author.

## References

[1] Chang K Barlaben A Farley S . Masson's tumor in the ulnar artery. J Vasc Surg. (2012) 56:223–5. doi: 10.1016/j.jvs.2012.01.010. PMID: 22387264

[2] Nakamura M Anzai T Ishimizu E Ashikawa K Inoshita A Takata Y . Intravascular papillary endothelial hyperplasia of the maxillary sinus extending into the contralateral nasal cavity. Eur Arch Otorhinolaryngol. (2024) 281:2749–53. doi: 10.1007/s00405-024-08499-y. PMID: 38502360 PMC11024052

[3] Hashimoto H Daimaru Y Enjoji M . Intravascular papillary endothelial hyperplasia. A clinicopathologic study of 91 cases. Am J Dermatopathol. (1983) 5:539–46. doi: 10.1097/00000372-198312000-00004. PMID: 6666836

[4] Vicensoto Moreira Milhan N Cavassini Torquato L Costa V Carvalho De Marco A Rodarte Carvalho Y Lia Anbinde A . A mixed form of intravascular papillary endothelial hyperplasia in an uncommon location: case and literature review. Dermatol Online J. (2018) 24:8. doi: 10.5070/D3242038112 29630155

[5] Hua HJ Wu J Yang QY Sun HR Fan QH Li H . Intravascular papillary endothelial hyperplasia of the stomach: report of a case. Zhonghua Bing Li Xue Za Zhi. (2022) 51:664–6. doi: 10.3760/cma.j.cn112151-20211208-00898. PMID: 35785842

[6] Manoranjan B Mann JA Joseph JT Kelly JJ . Intraventricular Masson tumor: case report and systematic review of primary intracranial intravascular papillary endothelial hyperplasia. J Neurosurg Sci. (2022) 66:420–4. doi: 10.23736/S0390-5616.21.05372-8. PMID: 34342194

[7] Eguchi T Nakaoka K Basugi A Arai G Hamada Y . Intravascular papillary endothelial hyperplasia in the mandible: a case report. J Int Med Res. (2020) 48:300060520972900. doi: 10.1177/0300060520972900. PMID: 33233959 PMC7705293

[8] Susini T Molino C Castiglione F Olivieri S . Masson's vegetant hemangioendothelioma arising in the uterine cervix during pregnancy: a case report. J Womens Health (Larchmt). (2010) 19:1759–62. doi: 10.1089/jwh.2010.1979. PMID: 20695814

[9] Mohammadyari F Dufan T Ahmed YK Dolati P . Masson tumor of the central nervous system: A case report and review of literature. Am J Case Rep. (2022) 23:e937597. doi: 10.12659/AJCR.937597. PMID: 36540012 PMC9793341

[10] Abdo M Farouk N E. Elshinawy W Mohamed Ahmed E A. Raafat M Husien Abdo W . Masson’s tumor as an uncommon cause of neck mass: a case presentation. Vasc Endovascular Surg. (2024) 58:405–9. doi: 10.1177/15385744231215102. PMID: 37962479 PMC10996301

[11] Clearkin KP Enzinger FM . Intravascular papillary endothelial hyperplasia. Arch Pathol Lab Med. (1976) 100:441–4. 947306

[12] Sherring K Foot O Barton DP Vroobel KM . Ovarian extravascular papillary endothelial hyperplasia (Masson Tumor) mimicking a primary gynecologic Malignancy. Int J Gynecol Pathol. (2021) 40:286–9. doi: 10.1097/PGP.0000000000000692. PMID: 32897969

[13] Agarwal S Gaur K Arya V . Intravascular papillary endothelial hyperplasia: olanzapine-induced vascular proliferation? Indian J Pathol Microbiol. (2023) 66:366–8. doi: 10.4103/ijpm.ijpm_356_21. PMID: 37077086

[14] Levere SM Barsky SH Meals RA . Intravascular papillary endothelial hyperplasia: a neoplastic “actor” representing an exaggerated attempt at recanalization mediated by basic fibroblast growth factor. J Handb Surg Am. (1994) 19:559–64. doi: 10.1016/0363-5023(94)90256-9. PMID: 7963307

[15] Rizza V Coletti G Cocco DP Mazzotta C Famulari A Pisani F . Serious renal hemorrhage in Masson tumor. Transplant Proc. (2009) 41:1402–4. doi: 10.1016/j.transproceed.2009.03.010. PMID: 19460571

[16] Sieiński W . Tumor-like intravascular proliferations of the stroma in adenomyosis. Patol Pol. (1993) 44:1–4 8488076

[17] Wagh VB Kyprianou I Burns J Brown LJR Vaidhyanath R Sampath R . Periorbital Masson’s tumor: a case series. Ophthalmic Plast Reconstr Surg. (2011) 27:e55–7. doi: 10.1097/IOP.0b013e3181e978f4. PMID: 20829727

[18] Kugler A Koelblinger P Zelger B Ahlgrimm-Siess V Laimer M . Papillary intralymphatic angioendothelioma (PILA), also referred to as Dabska tumour, in an 83-year-old woman. J Eur Acad Dermatol Venereol. (2016) 30:e59–61. doi: 10.1111/jdv.13300. PMID: 26333144

[19] Anthony SG Mudgal CS DeLaney TF Shin RD Raskin KA Ring DC . Recurrent intravascular papillary endothelial hyperplasia of the right middle finger treated with radiation therapy. J Bone Joint Surg Br. (2008) 90:95–7. doi: 10.1302/0301-620X.90B1.19726. PMID: 18160508

[20] Ng HJH Sio BR Desai V Chew KM Rajaratnam V . Intravascular papillary endothelial hyperplasia: case report of a recurrent Masson’s tumor of the finger and review of literature. J Handb Microsurg. (2021) 13:164–8. doi: 10.1055/s-0039-3401381. PMID: 34602798 PMC8463135

[21] Anghileri E Pollo B Ferroli P Aquino D Demichelis G Schiariti M . Case report: multiple brain intravascular papillary endothelial hyperplasia: incidence, diagnostic challenges, and management approach. Front Neurol. (2023) 14:1115325. doi: 10.3389/fneur.2023.1115325. PMID: 37153668 PMC10157200

